# Assessment of animal management and habitat characteristics associated with social behavior in bottlenose dolphins across zoological facilities

**DOI:** 10.1371/journal.pone.0253732

**Published:** 2021-08-30

**Authors:** Lance J. Miller, Lisa K. Lauderdale, Jill D. Mellen, Michael T. Walsh, Douglas A. Granger

**Affiliations:** 1 Conservation Science and Animal Welfare Research, Chicago Zoological Society–Brookfield Zoo, Brookfield, IL, United States of America; 2 Biology Department, Portland State University, Portland, OR, United States of America; 3 Department of Comparative, Diagnostic & Population Medicine, College of Veterinary Medicine, University of Florida, Gainesville, FL, United States of America; 4 Institute for interdisciplinary Salivary Bioscience Research, University of California, Irvine, CA, United States of America; Laboratoire d’Ethologie Experimentale et Comparee, FRANCE

## Abstract

Bottlenose dolphins are a behaviorally complex, social species that display a variety of social behaviors. Because of this, it is important for zoological facilities to strive to ensure animals display species-appropriate levels of social behavior. The current study is part of the multi-institutional study entitled “Towards understanding the welfare of cetaceans in zoos and aquariums” commonly referred to as the Cetacean Welfare Study. All participating facilities were accredited by the Alliance of Marine Mammal Parks and Aquariums and/or the Association of Zoos and Aquariums. Behavioral data were collected on 47 bottlenose dolphins representing two subspecies, *Tursiops truncatus* and *Tursiops aduncus*, at 25 facilities. The social behaviors of group related activity (*group active*) as well as interacting with conspecifics (*interact with conspecific*) were examined for their relationships to both animal management factors and habitat characteristics. The behavioral state of *group active* and the rate of *interact with conspecific* were both positively related to the frequency of receiving new forms of environmental enrichment. Both were inversely related to the random scheduling of environmental enrichment. Additional results suggested *interact with conspecific* was inversely related with daytime spatial experience and that males displayed *group active* more than females. Overall, the results suggested that animal management techniques such as the type and timing of enrichment may be more important to enhance social behavior than habitat characteristics or the size of the habitat. Information gained from this study can help facilities with bottlenose dolphins manage their enrichment programs in relation to social behaviors.

## Introduction

Common bottlenose dolphins (*Tursiops truncatus*) and Indo-Pacific bottlenose dolphins (*Tursiops aduncus*) are a behaviorally complex, social species that live in a fission-fusion society. These highly dynamic societies can change in both composition and structure throughout the day [1]. While typically found in groups ranging from a few to a dozen in coastal and estuarine habitats, larger groups can be found in open water [1–3]. Given their fission-fusion society, it is not surprising that they can spend anywhere between 4% and 31% of their time socializing [4–8]. In two of the most studied populations, Sarasota Bay Florida and Shark Bay Australia, females typically socialize with other females in their groups [9–11]. Male bottlenose dolphins on the other hand usually form strong bonds with other males in order to form an alliance [9,10,12]. These social behaviors and bonds appear to be important to these species.

In the wild there are many benefits to group living for bottlenose dolphins including protection from predators and cooperative hunting [13,14]. However, dolphins under professional care receive a readily available high-quality diet and are not exposed to potential predators but likely receive other benefits from group living [15]. Research has shown that strong social bonds can have adaptive consequences. For example, humpback whales (*Megaptera novaeangliae*) with stable and strong relationships have the highest reproductive success [16]. For many primate species, strong social bonds are associated with males siring more offspring [17], and females having higher reproductive success and longer lifespans [18–20]. Similarly, strong bonds between bottlenose dolphins also result in higher reproductive success [21,22]. It is likely that these social relationships buffer the deleterious effects of stress and thus provide adaptive value. Determining animal care and management factors that lead to positive social relationships may enhance the welfare of animals under professional care [23,24].

The social behavior of bottlenose dolphins under professional care has many similarities to the behavior of their wild counterparts [25]. For example, many of the affiliative behaviors such as pectoral fin touching are often seen under multiple populations in professional care [25,26]. However, some differences exist between wild and professionally managed dolphins due to habitat and animal management characteristics impacting social behavior. One study demonstrated that by changing habitat from a closed environment to an open “ocean pen” environment there was a decrease in social interaction for bottlenose dolphins [27]. The total size of the open environment was approximately five times greater than the capacity of the closed environment which were divided into three versus four areas respectively. The resulting change in size could account for the decrease observed, or the change could be due to dolphins in the open environment spending more time exploring the environment that would likely have other forms of wildlife.

Alternatively, participation in interaction programs resulted in an increase in common dolphins (*Delphinus delphis*) touching in a non-aggressive manner [28]. Additional studies have examined how interaction programs impacted the social behavior of bottlenose dolphins finding no differences comparing before and after the programs [29–31]. Similarly, there was no difference observed in social behavior before and after dolphin presentations [30]. These results suggest that these types of educational programs have a neutral or possibly a positive effect on dolphin social behavior.

Adding to the importance of social relationships for bottlenose dolphins, previous research has shown that when dolphins are given the choice between enrichment objects and social interactions, the latter was frequently selected [32]. This is not to imply that environmental enrichment is not important for dolphins, but to highlight the importance of social behavior for these species. In fact, multiple studies have shown that providing enrichment in the form of cognitive challenges increased social behavior [33,34]. However, those studies also resulted in the animals receiving a food reward making it is difficult to determine the primary factor responsible for impacting social behavior.

The goal of the current study was to examine factors associated with higher levels of social behavior for bottlenose dolphins under professional care. Specifically, environmental enrichment, animal training, and exhibit characteristics were examined to identify their relationship with social behavior for common bottlenose dolphins and Indo-Pacific bottlenose dolphins.

## Methods

### Ethics statement

The project was reviewed and approved by veterinary and animal care staff at each zoological facility. The U.S. Navy Marine Mammal Program Institutional Animal Care and Use Committee also reviewed and approved this research project under proposal #123–2017.

### Subjects and facilities

Focal animals for the project included common bottlenose dolphins and Indo-Pacific bottlenose dolphins. Facilities caring for the focal subspecies that were accredited by either the Alliance of Marine Mammal Parks and Aquariums (AMMPA) or Association of Zoos and Aquariums (AZA) were invited to participate. The current study is one component of a larger study entitled “Towards Understanding the Welfare of Cetaceans in Zoos and Aquariums”. We used a semi-random sample design to select two animals from each facility accounting for sex and age. This was done to have a sample representative of the variability in dolphin age and sex across facilities. Data were collected on 86 dolphins from 40 facilities during two different five-week periods. Data were collected on six dolphins during the first five-week period but not the second five-week period. Data from eight dolphins were collected during the second five-week period that were not part of the first five-week period. Data were also collected on two dolphins that changed facilities between the two five-week periods. S4 Appendix 1 in S4 File lists all dolphins that were part of the study including sex, age, and total minutes visible.

### Data collection

Data were collected during two five-week periods between July and November of 2018 and from January through April of 2019. During each of the five-week periods data were collected by videotaping focal animals three times a week. Videotaping occurred during one of three time periods: morning (8:00–11:00), mid-day (11:00–14:00), and afternoon (14:00–17:00). Animal care staff or interns at each facility were requested to videotape the animals to ensure accurate identification of the animals. Table 1 displays the counterbalanced recording schedule for all facilities across dolphins, day, and time periods. Animal care staff and interns were allowed to videotape at any point within the assigned time period. Videographers were instructed to not film within 20 minutes before or following a dolphin presentation, interaction program, training session, or research session. Before data collection began at each facility, the videographer spent a week (based on the schedule) standing in the filming location to habituate the dolphins to the person filming. They were also informed not to interact (eye contact or engaging with) with any of the animals while recording. The name of the dolphin, exhibit code, time, date, identification number, and observation numbers were written on a piece of paper and held in frame before each observation began. Observations were 25 minutes in length resulting in a total of 375 minutes of data during each five-week data collection period of the study. A Fuji Film XP120 waterproof video camera with a polarizing filter was used reduce glare. To ensure data were comparable across facilities, recording was done from above water because not all facilities had underwater viewing.

**Table 1:** Observation schedule used to record focal animals across all facilities.

Week	Time Period	Monday	Tuesday	Wednesday	Thursday	Friday	Saturday	Sunday
1	8:00–11:00	D1			D2			
	11:00–14:00	D2		D1				
	14:00–17:00			D2	D1			
2	8:00–11:00	D2					D1	
	11:00–14:00						D2	D1
	14:00–17:00	D1						D2
3	8:00–11:00			D1			D2	
	11:00–14:00			D2	D1			
	14:00–17:00				D2		D1	
4	8:00–11:00			D2				D1
	11:00–14:00	D1						D2
	14:00–17:00	D2		D1				
5	8:00–11:00				D1			D2
	11:00–14:00				D2		D1	
	14:00–17:00						D2	D1

Note: D1 is Focal Dolphin 1 and D2 is Focal Dolphin 2. All trained observers were required to reach reliability of r > 0.80 compared to a primary observer before actual scoring of behavior from the video occurred. Nine observers reached reliability and were utilized to score videos. Video was scored by using a combination of continuous sampling (behavioral events) and instantaneous sampling (behavioral states). Scans for instantaneous sampling were one minute apart resulting in 25 scans per video. While additional behaviors were scored as part of the larger study, the current study focuses primarily on two types of social behavior. These include the behavioral state of group related activity (*group active*) as well as the behavioral event of interacting with conspecifics (*interact with conspecific*). *Group active* was defined as a dolphin engaged with at least one other dolphin with no sustained forward-only movement (e.g., group swimming) which included rubbing, chasing, mounting, or jumping. This was a state coded based on the dolphin’s swim pattern/type. However, this behavior does not provide information on individual behaviors that were displayed while in the swim type. *Interact with conspecific* was defined as a dolphin orienting towards and mutually interacting with one or more conspecifics for more than 3 s. *Interact with conspecific* was considered to be a specific behavior and did not include all forms of social behavior. The main difference in scoring between the two variables is that one was a behavioral state and the other was a behavioral event. As aggressive behavior was included in the main social behaviors of interest, descriptive statistics are also included for potentially aggressive behaviors recorded during the study. These included bite/rake, fluke slap, pectoral slap, and chin slap. Bite/rake was defined as a dolphin forcefully closes mouth around another dolphin or forceful contact with another dolphin by rubbing/sliding its jaws on the other dolphin. This can include a pectoral fin, fluke, or other body part. Fluke slap was defined as a dolphin makes sharp/smacking contact with its fluke to the surface of the water. Pectoral slap was defined as a dolphin smacks or slaps its pectoral fin on the surface of the water, and chin slap was defined as a dolphin lifts head from water and smacks or slaps the surface of the water with the lower jaw. Definitions for social behavior were adapted from previous studies [3,30,35–40]. *Group active* was converted to percentage of visible scans and *interact with conspecific* was converted to a rate by dividing by total minutes visible.

As previously noted, the cameras were fixed with polarizing filters to reduce the glare. However, for some outdoor facilities the glare was too intense, and dolphins were not visible for long periods of time. In addition, for some of the open ocean facilities animals were also not visible for long periods of time due to turbidity changes from particulate, algae, or plankton. In order to ensure reliable and valid data, for each dolphin, data were analyzed for either the first or second five-week data collection period, using a criterion of at least 240 minutes of visibility. The criterion of 240 minutes was chosen as it would have required at least 10 observations over the five-week period and the animals would have been required to be visible for the majority of the total possible time filmed. Dolphins that were visible less than 240 minutes during both the first and second five-week data collection period were dropped from further analysis. Dolphins with more than 240 minutes total time visible during both five-week data collection periods had the second period dropped from further analysis. The second data collection period was dropped in this scenario even when meeting the 240-minute criteria due to statistical analysis excluding rows with missing data. Finally, dolphins with more than 240 minutes for only the first five-week data collection period were retained for analysis. A single five-week data collection period was selected because dolphins without data in both five-week periods would have been excluded entirely when building the generalized estimating equation (GEE) models, further reducing the sample size. The decision resulted in the largest possible sample size to investigate variability across facilities rather than within individual dolphins. A chi-square test of significance was used to ensure resulting sample size was not significantly different from original sample based on sex or habitat type and an independent t-test was used to ensure there were no differences based on age.

### Independent variables

Independent variables were selected to examine a variety of animal care and management factors as well as exhibit characteristics that may be associated with social behavior. These variables were created from a survey that was sent to each facility to detail their habitat characteristics, environmental enrichment program, and animal training routine for the dolphins examined in this study. The full list of independent variables can be found in Table 2 along with definitions. Any independent variables that were calculated from the management survey and not direct data are detailed by [41].

**Table 2:** Independent variables included in the analysis.

Variable	Definition	Values	Type of Variable
***Demographic***			
Sex	Sex of the dolphin	Male/Female	Factor
Age	Age of the dolphin	Years	Covariate
***Environmental Enrichment***			
Enrichment Diversity Index	Enrichment diversity index was created using the Shannon diversity index on the mean number of days each enrichment is provided at the facility	Index	Covariate
Enrichment Program Index	Enrichment program index is a standardized factor score created with scores on frequency of enrichment program components used at the facility using a polychoric PCA	Index	Covariate
Night Time Enrichment	Mean number of nights in a week that enrichment was provided to the dolphins at the facility	Number of Nights	Covariate
Enrichment Schedule	Categorical value indicating how enrichment was scheduled at the facility	Predictable, Semi-Random, Random	Factor
Frequency of New Enrichment	Categorical frequency that a facility provided the dolphins with new types/forms of enrichment	Weekly/Monthly, Twice a Year, Yearly/Year+	Factor
***Training***			
Dolphin Presentations	Mean number of dolphin presentations an individual dolphin participated in each week	Mean Number of Presentations	Covariate
Interaction Programs	Mean number of dolphin interaction programs an individual dolphin participated in each week	Mean Number of Interactions	Covariate
Training Duration	Mean amount of time each dolphin interacted with an animal care professional for presentations, interaction programs, training sessions, research, or other training activities each week	Hours	Covariate
Maximum Number of Interaction Guests	Maximum number of participants allowed for an interaction program for that facility	Number of Participants	Covariate
Training Schedule	Categorical variable indicating if the training schedule for the dolphins at that facility was predictable or semi-predictable	Predictable, Semi-Predictable	Factor
***Habitat Characteristics***			
Day Time Spatial Experience	Proportionate volume of water the dolphin had access to based on the percentage of daytime hours spent in different habitats in each five-week data collection period	Megaliter	Covariate
Night Time Spatial Experience	Proportionate volume of water the dolphin had access to based on the percentage of nighttime hours spent in different habitats in each five-week data collection period	Megaliter	Covariate
24 Hour Spatial Experience	Proportionate volume of water the dolphin had access to based on the percentage of hours throughout the entire day spent in different habitats in each five-week data collection period	Megaliter	Covariate
Length	The maximum straight length in any direction across any habitat the dolphin had access to in each five-week data collection period	m	Covariate
Depth	The maximum depth for any habitat the dolphin had access to in each five-week data collection period	m	Covariate
Habitat Type	Categorical variable indicating the dolphin was in a professionally managed zoo/aquarium habitat or a professionally managed ocean habitat	Zoo/Aquarium, Ocean	Factor
Number of Habitats	Maximum number of habitats (different enclosures) dolphin had access to in daytime hours during each five-week data collection period	Number of Habitats	Covariate
Social Management	Categorical variable indicating the type of social management practice for a dolphin during each five-week data collection period	Same Group, Split/Reunited, Rotated Subgroups	Factor
Neighboring Conspecifics	Categorical variable indicating if the dolphin had visual and auditory access to other dolphins without possibility of physical contact during each five-week data collection period	No, Yes	Factor

### Statistical analysis

All descriptive statistics for independent and dependent variables can be found in Supplementary Document S1 in S1 File. All statistical models were examined using GEE given the non-normal distribution of the data in SPSS Version 27. GEE benefits analysis when variables are not normally distributed as they do not require transformations which makes interpretation of results easier [42,43]. Individual dolphin was the unit of analysis for all models while controlling for facility as a within-subject variable and any significant demographic variables (age and sex). Step one of building the models required examining univariate relationships and only variables with p < 0.15 were used to develop and examine the multivariate models [44,45]. Final models were selected based on the best quasi-likelihood under the independence model criterion (QIC) and significance for independent variables [44,46]. The final models that were considered with significant independent variables and the lowest QIC values are in Supplementary Document S2 in S2 File.

## Results

Based on the minimum criteria of being visible for greater than 240 minutes, the final sample size included 47 dolphins from 25 accredited zoological facilities. Dolphins ranged from 4 to 47 years of age (average 19.68 ± 12.39 SD). The resulting sample size was not statistically different from the original sample size based on sex (χ^2^(1, N = 133) = 0.01, *p* > 0.05), age (t (131) = -1.105, *p* > 0.05), or habitat type (χ^2^(1, N = 133) = 3.016, *p* > 0.05). There was a total of 43 common bottlenose dolphins (91.3%) and 4 Indo-pacific bottlenose dolphins (8.7%) with 28 males and 19 females. Across all individuals throughout the five-week period, *group active* ranged from 0.00 to 33.79 percent of visible scans while *interact with conspecific* ranged from a rate of 0.00 to 0.72 per minute visible. Table 3 highlights the rates of aggressive behavior observed throughout the study.

**Table 3:** Descriptive statistics highlighting the rates of aggression recorded during the study.

Behavior	Mean	Median	Standard Deviation
Bite/Rake	0.000	0.000	0.003
Fluke Slap	0.012	0.000	0.041
Pectoral Slap	0.001	0.000	0.003
Chin Slap	0.000	0.000	0.012

Following univariate analysis (Tables 4 and 5), eight variables were considered for multivariate models for *group active* and eight variables were considered for multivariate models for *interact with conspecific*. Descriptive statistics for all independent variables considered for multivariate models are listed in Tables 6 and 7.

**Table 4:** Summary of univariate analysis for relationships between independent variables and *group active*.

Variables	Reference	n	Beta	*p*-value
***Demographic***				
Sex	Ref = Male	28	0.000	
	Female	19	-0.360	0.032*
Age		47	-0.002	0.001*
***Environmental Enrichment***				
Enrichment Diversity Index		47	0.031	0.020*
Enrichment Program Index		47	-0.001	0.929
Night Time Enrichment		47	0.001	0.704
Enrichment Schedule	Ref = Predictable	14	0.000	
	Semi-Random	28	0.014	0.480
	Random	5	-0.027	0.082^
Frequency New Enrichment	Ref = Year+/Yearly	7	0.000	
	Twice a Year	14	0.058	0.000*
	Monthly/Weekly	28	0.045	0.004*
***Training***				
Dolphin Presentations		47	-0.001	0.448
Interaction Programs		47	0.000	0.851
Training Duration		47	-0.003	0.116^
Maximum Number Interaction Guests		47	-0.001	0.268
Training Schedule	Ref = Predictable	16	0.000	
	Semi-Predictable	31	-0.008	0.730
***Habitat Characteristics***				
Day Time Spatial Experience		47	-0.008	0.491
Night Time Spatial Experience		47	0.001	0.890
24 Hour Spatial Experience		47	-0.007	0.533
Length		47	0.000	0.581
Depth		47	0.001	0.657
Habitat Type	Ref = Zoo/Aquarium	36	0.000	
	Ocean	11	-0.031	0.117^
Number of Habitats		47	0.005	0.155
Social Management	Ref = Same Group	17	0.000	
	Split/Reunited	21	0.020	0.269
	Rotated Subgroups	9	0.071	0.003*
Neighboring Conspecifics	Ref = No Visual Access	31	0.000	
	Visual/Auditory Access	16	-0.001	0.947

^*p* value < 0.15 utilized as significance level for variable selection

*p value < 0.05.

**Table 5:** Summary of univariate analysis for relationships between independent variables and *interact with conspecific*.

Variables	Reference	N	Beta	*p*-value
***Demographic***				
Sex	Ref = Male	28	0.000	
	Female	19	-0.056	0.100^
Age		47	-0.002	0.213
***Environmental Enrichment***				
Enrichment Diversity Index		47	0.035	0.102^
Enrichment Program Index		47	-0.022	0.188
Night Time Enrichment		47	0.006	0.365
Enrichment Schedule	Ref = Predictable	14	0.000	
	Semi-Random	28	0.047	0.228
	Random	5	-0.043	0.116^
Frequency New Enrichment	Ref = Year+/Yearly	7	0.000	
	Twice a Year	14	0.102	0.001*
	Monthly/Weekly	28	0.109	0.001*
***Training***				
Dolphin Presentations		47	-0.003	0.153
Interaction Programs		47	0.000	0.998
Training Duration		47	-0.007	0.150
Maximum Number Interaction Guests		47	-0.003	0.106^
Training Schedule	Ref = Predictable	16	0.000	
	Semi-Predictable	31	-0.017	0.638
***Habitat Characteristics***				
Day Time Spatial Experience		47	-0.053	0.085^
Night Time Spatial Experience		47	-0.010	0.651
24 Hour Spatial Experience		47	-0.040	0.193
Length		47	-0.003	0.136^
Depth		47	-0.003	0.401
Habitat Type	Ref = Zoo/Aquarium	36	0.000	
	Ocean	11	0.000	0.997
Number of Habitats		47	0.009	0.250
Social Management	Ref = Same Group	17	0.000	
	Split/Reunited	21	0.084	0.035*
	Rotated Subgroups	9	0.105	0.001*
Neighboring Conspecifics	Ref = No Visual Access	31	0.000	
	Visual/Auditory Access	16	0.019	0.682

^*p* value < 0.15 utilized as significance level for variable selection

*p value < 0.05.

**Table 6:** Descriptive statistics for independent variables considered for multivariate model of *group active*.

Independent Variable	Reference	N	Mean	SD	Min	Max	Median
Sex	Ref = Male	28	-	-	-	-	-
	Female	19	-	-	-	-	-
Age	-	47	19.68	12.45	4.00	47.00	15.00
Enrichment Diversity Index	-	47	1.74	0.65	0.00	2.59	1.89
Enrichment Schedule	Ref = Predictable	14	-	-	-	-	-
	Semi-Random	28	-	-	-	-	-
	Random	5	-	-	-	-	-
Frequency New Enrichment	Ref = Year+/Yearly	7	-	-	-	-	-
	Twice a Year	14	-	-	-	-	-
	Monthly/Weekly	28	-	-	-	-	-
Training Duration (h)		47	674.50	266.85	171.20	1489.00	635.80
Habitat Type	Ref = Zoo/Aquarium	36					
	Ocean	11					
Social Management	Ref = Same Group	17	-	-	-	-	-
	Reunited	21	-	-	-	-	-
	Rotated Subgroups	9	-	-	-	-	-

**Table 7:** Descriptive statistics for independent variables considered for multivariate model of *interact with conspecific*.

Independent Variable	Reference	n	Mean	SD	Min	Max	Median
Sex	Ref = Male	28	-	-	-	-	-
	Female	19	-	-	-	-	-
Enrichment Diversity Index	-	47	1.74	0.65	0.00	2.59	1.89
Enrichment Schedule	Ref–Predictable	14	-	-	-	-	-
	Semi-Random	28	-	-	-	-	-
	Random	5	-	-	-	-	-
Frequency New Enrichment	Ref = Year+/Yearly	7	-	-	-	-	-
	Twice a Year	14	-	-	-	-	-
	Monthly/Weekly	28	-	-	-	-	-
Maximum Number Interaction Guests		47	10.72	10.23	0.00	30.00	10.00
Day Time Spatial Experience (ML)		47	1.15	0.62	0.12	2.39	0.91
Length (m)		47	35.76	12.61	9.14	66.00	35.60
Social Management	Ref = Same Group	17	-	-	-	-	-
	Reunited	21	-	-	-	-	-
	Rotated Subgroups	9	-	-	-	-	-

Multivariate models for *group active* were examined using sex and age (demographic), enrichment diversity index, enrichment schedule, and frequency of new enrichment (environmental enrichment), training duration (animal training), habitat type, and social management (habitat characteristics). Multivariate models for *interact with conspecific* were examined using sex (demographic), enrichment diversity index, enrichment schedule, and frequency of new enrichment (environmental enrichment), maximum number of interaction guests (animal training), day time spatial experience, length of habitat, and social management (habitat characteristics). The final multivariate model for *group active* included sex, enrichment schedule, and frequency of new enrichment (Table 8). Female dolphins displayed *group active* less when compared to male dolphins (β = -0.036, *p* = 0.017). Dolphins that were provided enrichment on a random schedule displayed *group active* less than animals provided enrichment on a predictable schedule (β = -0.053, *p* = 0.002). Additionally, dolphins that were provided new forms of enrichment twice per year or monthly/weekly displayed *group active* more than animals provided new forms of enrichment annually or less frequently (twice per year: β = 0.065, *p* < 0.001; monthly/weekly: β = 0.055, *p* = 0.002). The final multivariate model for *interact with conspecific* included enrichment schedule, frequency of new enrichment, and day time spatial experience (Table 9). Similar to *group active*, dolphins that were provided enrichment on a random schedule *interact with conspecific* less than animals provided enrichment on a predictable schedule (β = -0.078, *p* = 0.025). Also similar to *group active*, dolphins that were provided new forms of enrichment twice per year or monthly/weekly *interact with conspecific* more than animals provided new forms of enrichment annually or less frequently (twice per year: β = 0.115, *p* = 0.002; monthly/weekly: β = 0.118, *p* = 0.003). Finally, there was an inverse relationship found between *interact with conspecific* and daytime spatial experience β = -0.057, *p* = 0.045).

**Table 8:** Results from multivariate model examining *group active* (*p < 0.05).

Variable	Beta	95% Confidence Interval	*p*-value
Intercept	0.035	0.012–0.058	0.003*
Sex	-0.036	-0.066–-0.007	0.017*
Enrichment Schedule: Predictable	-		
Enrichment Schedule: Semi-Random	-0.009	-0.041–0.022	0.564
Enrichment Schedule: Random	-0.053	-0.086–-0.020	0.002*
Frequency of New Enrichment: Yearly/Yearly+	-		
Frequency of New Enrichment: Twice Yearly	0.065	0.032–0.098	<0.001*
Frequency of New Enrichment: Monthly/Weekly	0.055	0.020–0.090	0.002*

**Table 9:** Results from multivariate model examining interaction with conspecific (*p < 0.05).

Variable	Beta	95% Confidence Interval	*p*-value
Intercept	0.084	0.010–0.158	0.026*
Enrichment Schedule: Predictable	-		
Enrichment Schedule: Semi-Random	0.018	-0.056–0.092	0.640
Enrichment Schedule: Random	-0.078	-0.147–-0.010	0.025*
Frequency of New Enrichment: Yearly/Yearly+	-		
Frequency of New Enrichment: Twice Yearly	0.115	0.041–0.189	0.002*
Frequency of New Enrichment: Monthly/Weekly	0.118	0.039–0.197	0.003*
Day Time Spatial Experience	-0.057	-0.112–-0.001	0.045*

## Discussion

It is important to understand factors that are associated with species-appropriate levels of social behavior for bottlenose dolphins under professional care. In the current study, the behaviors of *group active* and *interact with conspecific* were used to explore factors that are associated with social behavior. Given the low levels of aggression observed throughout the study, the factors associated with *group active* and *interact with conspecific* are associated with primarily affiliative behavior in bottlenose dolphins. Additionally, previous research has suggested that tail slaps, one of the behaviors listed as aggressive in the current study, may not always be a form of aggression similar to an animal breaching [47]. While female dolphins displayed *group active* less than males, and day time spatial experience was inversely related to *interact with conspecific*, the majority of significant predictors were variables associated with environmental enrichment. These included both the timing of enrichment as well as the frequency with which animals were provided with new forms of environmental enrichment.

In the current study, the timing of environmental enrichment refers to the randomness in temporal predictability. Dolphins on a more predictable schedule were more likely to display both group active and *interact with conspecific* compared to dolphins provided enrichment on a random schedule. Previous research on the predictability of receiving environmental enrichment for animals under professional care is scarce. More research has been conducted on the temporal predictability of receiving food, another positive experience in the care of animals. A review of the research on predictability of favorable events suggests that a random temporal or unpredictable schedule would be best if cues as to when food would be provided were predictable [48]. However, those authors note that this has been studied across few taxonomic groups and could differ depending on the species. The level of predictability in receiving food in relation to activity levels and behavioral diversity was observed in fennec fox (*Vulpes zerda*) [49]. Results suggested that moderate levels of temporal predictability optimized both activity levels and behavioral diversity when compared to 100% predictable or 100% unpredictable food availability. This would suggest the semi-random schedule of enrichment might be optimal for dolphins, but was not the case in the current study for social behavior. Instead, a more predictable schedule was associated with higher levels of both *group active* and *interact with conspecific*. Additionally, a random schedule of enrichment also related to lower energy expenditure in bottlenose dolphins [50], which may be accounted for by the lower rates of *group active* and *interact with conspecific* observed in the current study. Moving forward, animal care specialists that care for bottlenose dolphins can explore moderate levels of randomization for enrichment to enhance social behavior, but a completely random schedule would not be advisable. Additionally, a recent study suggested that providing enrichment directly following training sessions may not be appropriate given the high levels of synchronous swimming observed [23]. This type of behavior has been demonstrated as beneficial for dolphin’s social bonds and welfare and providing enrichment could potentially interfere with these important behaviors [51,52]. If providing enrichment on a completely predictable schedule, it would be important for facilities to determine the best possible time based on the behavior of those specific animals.

In contrast, providing new forms of enrichment on weekly/monthly or twice annual schedule was positively associated with both *group active* and *interact with conspecific* when compared to providing it yearly or less frequently. Previous research has shown that the frequency of providing new forms of enrichment can be just as important as the type of enrichment provided [53]. For example, pigs quickly habituated to objects existing within their enclosure, but exploratory behavior was maintained by consistently replacing old items with new items [54]. Recently, the frequency of providing new enrichment has also been shown to positively relate to energy expenditure, distance traveled, and behavioral diversity, all likely demonstrating positive welfare [50,55]. Given the intelligence of bottlenose dolphins [56], providing new forms of enrichment on a regular basis would likely be beneficial and promote positive welfare. Previous research has shown that dolphins display anticipatory behavior before receiving enrichment, and the amount of anticipatory behavior correlates with the duration of use of the enrichment [57]. This provides further support of the importance of environmental enrichment as anticipatory behavior can be an indicator of motivation [58].

The final relationships observed with *group active* and *interact with conspecific* included day time spatial experience and sex. Day time spatial experience was inversely related to *interact with conspecific* suggesting that dolphins interact more in smaller habitats. This relationship could potentially be explained by density of individuals, or having the dolphins in a larger habitat, decreasing the likelihood of interaction. Previous research has shown similar results, with bottlenose dolphins displaying less social behavior in larger habitats [27]. However, this change in social behavior could also be due to animals in the larger habitats spending more time exploring the environment in the open “ocean pen” that could have other forms of wildlife. We would not recommend creating smaller habitats to encourage appropriate levels of social behavior. Instead, it would be more important to focus on building strong social groups and bonds through the relationships observed in the current study with environmental enrichment.

Finally, females were less likely to display *group active* when compared to males. This could possibly be explained by the fact that male dolphins in the wild create complex lifelong alliances and affiliative social behavior would help in forming and maintaining those relationships [59,60]. An additional explanation could be that we only examined one form of swimming pattern in *group active*, and it is quite possible that if synchronous or group swimming had been included, the females would have been observed at higher levels. Previous research has determined this type of synchronous swimming is an important behavior for developing social bonds in dolphins [51,52]. Future research should also examine how habitat characteristics, environmental enrichment, and training programs impact other forms of social behavior. Given the current findings and fact that group composition was not examined in the current study, there are few recommendations we could make based on the relationship observed with sex.

It is clear that facilities and their staff that care for bottlenose dolphins can utilize enrichment strategies to enhance species-appropriate levels of social behavior such as *group active* or *interact with conspecific*. Previous research has found that cognitive enrichment [33] and other forms of environmental enrichment [61] increase levels of social play. However, the former study noted an increase in social play specifically when dolphins acquired a gelatin ball, so play could be a result of the puzzle or simply having a new consumable object. Similarly, engaging in a problem-solving task as a form of environmental enrichment increased social behavior for a group of bottlenose dolphins at another facility, but the animals also received a food reward as part of the puzzle [34]. However, another study which utilized a collaborative cognitive challenge with bottlenose dolphins found no impact on social behavior [62]. With the recommendation to find new ways of providing new forms of enrichment on a continuous basis, cognitive enrichment may be one way to facilitate being creative with an enrichment program. However, staff would be encouraged to examine the impact of any new enrichment for the animals under their professional care to ensure it is having a positive effect on their welfare.

Ultimately, ensuring that bottlenose dolphins under professional care have the ability to socialize at species-appropriate levels is important. Previous research has shown that dolphins that display more social affiliative behavior judge ambiguous cues more optimistically [51]. Ensuring that animals are in a positive emotional state is critical to the welfare of bottlenose dolphins under professional care. Moving forward, animal care staff can utilize aspects of enrichment programs found to relate to social behavior.

One of the limitations of the current study was the decrease in subjects as a result of issues with visibility. However, this is still the largest multi-institutional study on cetacean welfare. Additionally, we were unable to control for subspecies in the current analysis given the small number of *Tursiops aduncus*. However, given their close relatedness we do not believe this to be a significant limitation. The significant and non-significant relationships observed can help drive animal management decisions regarding the social behavior of bottlenose dolphins under professional care. Future research should examine additional factors related to ensuring species-appropriate levels of social behavior given the highly complex social nature of the species. Given the importance of social behavior, this in turn can help ensure that dolphins are experiencing positive welfare and can continue to inspire people to become engaged in conservation activities [63].

## Supporting information

S1 File Descriptive statistics Miller et al social behavior. (XLSX)

S2 File Model selection Miller et al social behavior. (XLSX)

S3 File Striking image Miller et al social behavior. (TIFF)

S4 File Appendix1 Miller et al social behavior. (XLSX)
