# Supplementary figures and images for: Assessment of animal management and habitat characteristics associated with social behavior in bottlenose dolphins across zoological facilities
Source: PLoS One. 2021 Aug 30;16(8):e0253732. doi: 10.1371/journal.pone.0253732 (PMC8405028; doi:10.1371/journal.pone.0253732)

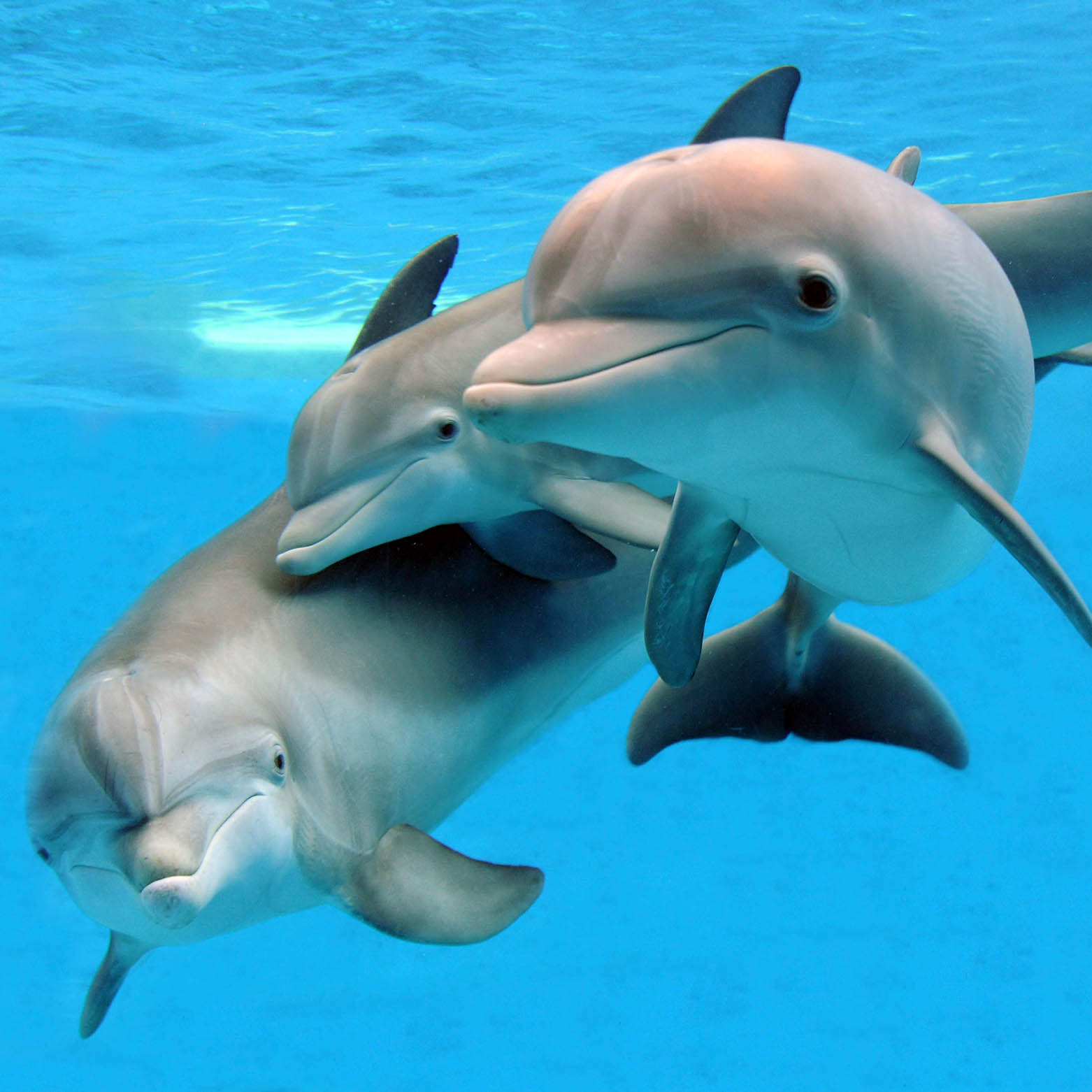

Supplement: S3 File — (TIFF) [file pone.0253732.s003.tiff]
